# Superiority of the ROMA index over HE4 and CA125 for diagnosing ovarian cancer in a Bangladeshi cohort

**DOI:** 10.1016/j.clinsp.2026.100927

**Published:** 2026-04-08

**Authors:** Pallab Kumar Das, Saddam Hossain, A.F.M. Ashrafur Rahman, Pritam Saha, Shaker Ahmed, Tauhid Md Mustakim, Kazi Fharia Tanjim, Asesh K. Chowdhury, Tawfika Rahman Jishan, Tanjina Tabassum

**Affiliations:** a Department of Microbiology, Pioneer Dental College & Hospital, Dhaka-1229, Bangladesh; b Department of Cardiology, Enam Medical College & Hospital, Dhaka-1340, Bangladesh; c Civil Surgeon Office, Brahmanbaria-3401, Bangladesh; d Projahnmo Research Foundation, Dhaka-1213, Bangladesh; e Dhaka Medical College Hospital, Dhaka-1100, Bangladesh; f Ukhiya Chittagong, Turkish Red Crescent, Bangladesh; g Diligent Dental, Mirpur, Dhaka-1216, Bangladesh; h Kidney Foundation Hospital, Mirpur, Dhaka-1216, Bangladesh; i Pi Research & Development Center, Dhaka-1100, Bangladesh; j Tropical Disease & Health Research Center, Dhaka-1100, Bangladesh

**Keywords:** Ovarian neoplasms, CA-125 antigen, WFDC2 protein, Human, Risk of ovarian malignancy algorithm, Biomarkers, Tumor

## Abstract

**Background:**

Ovarian cancer is a leading cause of mortality among women globally. This study aimed to evaluate the diagnostic value of CA-125, HE4, and the ROMA index in identifying ovarian cancer in patients in Bangladesh.

**Methods:**

This case-control study was conducted at the National Institute of Cancer Research and Hospital (NICRH) in 50 patients with histologically confirmed epithelial ovarian cancer, 25 patients with benign ovarian diseases, and 25 healthy controls. Women with prior chemotherapy or hormonal therapy, non-epithelial ovarian cancer, bilateral oophorectomy, pregnancy, or serious systemic illness were excluded. Serum CA-125 and HE4 levels were measured using Chemiluminescence Immunoassay (CLIA), and the ROMA index was calculated. Receiver Operating Characteristic (ROC) analysis was performed to assess diagnostic performance.

**Results:**

The mean ages of participants were 52.5-years for ovarian cancer patients, 38.2-years for those with benign diseases, and 45.0-years for healthy controls. The median serum levels of HE4, CA-125, and ROMA index were significantly higher in ovarian cancer patients compared to controls. ROC analysis revealed AUCs of 0.95 for ROMA, 0.92 for HE4, and 0.88 for CA-125 in differentiating cancer from healthy controls, and 0.93 for ROMA, 0.94 for HE4, and 0.60 for CA-125 in differentiating cancer from benign ovarian diseases. ROMA and HE4 significantly distinguished ovarian cancer from benign conditions (*p* < 0.001), while CA-125 did not (*p* = 0.165).

**Conclusion:**

In this study, the ROMA index appeared to perform better than HE4 and CA-125, though findings should be interpreted cautiously, considering the limited statistical power.

## Highlights

• ROMA showed higher accuracy (AUC: 0.93–0.95) than HE4 and CA-125 for ovarian cancer.
• ROMA, HE4 discriminated cancer vs benign (AUC ≥ 0.93), unlike CA-125 (0.60).
• ROMA validated as a specific triage tool for ovarian cancer in low-resource settings.
• Study provides key biomarker performance data from a Bangladeshi cohort.

## Introduction

Ovarian cancer remains one of the leading causes of cancer-related mortality among women worldwide, with a disproportionately higher burden in low- and middle-income countries.^1^ According to recent global estimates, approximately 300,000 new cases of ovarian cancer are diagnosed annually, resulting in over 180,000 deaths.^1^, ^2^, ^3^ This persistently high mortality-to-incidence ratio is largely attributable to delayed diagnoses, as early-stage ovarian cancer typically presents with nonspecific or absent symptoms that often mimic benign gynecological conditions. Additionally, there is a lack of an effective screening procedure for this disease.^4^ Consequently, there is a pressing clinical need to develop reliable, accessible, and cost-effective diagnostic tools for early detection of ovarian cancer, particularly in low-resource settings.

In current clinical practice, Carbohydrate Antigen 125 (CA-125) is the most commonly used tumor marker in ovarian cancer.^5^ Despite its widespread use, the sensitivity and specificity of this biomarker for the detection of ovarian cancer is not satisfactory.^6^ Elevated levels of CA-125 can also be found in other malignancies (e.g., endometrial, endocervical, lung, and pancreatic cancers) as well as in benign conditions (e.g., ovarian cysts, myomas, and endometriosis).^7^, ^8^, ^9^, ^10^, ^11^ Furthermore, the sensitivity of CA-125 varies with the tumor stages, demonstrating high sensitivity in advanced ovarian cancer cases but markedly reduced sensitivity in early-stage disease, where early intervention would offer the greatest survival benefit. This diagnostic limitation has been a significant obstacle in improving ovarian cancer outcomes and underscores the importance of incorporating additional biomarkers or diagnostic strategies capable of identifying malignancy at an earlier stage.^6^^,11^

To address the limitations of CA-125, several additional markers have been investigated to improve diagnostic accuracy, especially in early-stage ovarian cancer. Among these, Human Epididymis Protein 4 (HE4) has emerged as a potential biomarker due to its restricted expression in the reproductive tracts and respiratory epithelium, and its frequent overexpression in epithelial ovarian cancer.^12^ While HE4 levels naturally increase with age and are higher in postmenopausal women,^13^ they are significantly elevated in patients with ovarian cancer, especially those with serous and endometrioid histologies, but not in women with benign gynecological conditions or endometriosis.^14^^,^^15^ As a result, HE4 has demonstrated superior specificity over CA-125 and improved sensitivity for detecting ovarian cancer, both in early stages and in monitoring treated patients.^12^^,^^16^^,^^17^ Furthermore, HE4 has shown minimal cross-reactivity with benign gynecological conditions, making it a particularly valuable biomarker in clinical scenarios where CA-125 results might be ambiguous.

Recognizing the complementary strengths of CA-125 and HE4, recent clinical strategies have integrated these markers into the Risk of Ovarian Malignancy Algorithm (ROMA).^17^ ROMA calculates a predictive score for ovarian malignancy by integrating serum levels of CA-125 and HE4 with menopausal status.^18^ Its principal clinical advantage lies in its ability to improve early detection rates and better discriminate between benign and malignant pelvic masses, particularly in premenopausal women and those with early-stage disease, where CA-125 alone may be insufficient. In addition to enhancing diagnostic accuracy, ROMA offers clinicians a practical and interpretable tool that can guide patient triage decisions, optimize referral pathways, and prioritize surgical interventions, especially in resource-constrained healthcare systems.^19^

In healthcare settings like Bangladesh, histopathological examinations, the gold standard for ovarian cancer diagnosis, are often delayed or inaccessible due to the limited availability of advanced medical facilities and diagnostic tools. In this context, the utilization of tumor biomarkers such as CA-125 and HE4, along with clinical algorithms like ROMA, presents a practical alternative for early detection and risk stratification of ovarian malignancy. Despite the growing global evidence supporting these biomarkers, data on their diagnostic performance in the patient population of Bangladesh remain scarce, limiting their integration into routine clinical practice. Therefore, the authors hypothesize that the Risk of Ovarian Malignancy Algorithm (ROMA) will demonstrate superior diagnostic accuracy compared to HE4 and CA-125 serum levels alone for differentiating between benign and malignant ovarian masses. The primary objective of this study was to assess and compare the serum levels of CA-125, HE4, and ROMA scores in patients with ovarian cancer and those with benign pelvic masses in patients from Bangladesh. Additionally, the study aimed to evaluate the diagnostic accuracy of each biomarker individually and in combination to determine their clinical utility for early detection of ovarian malignancy in this population.

## Methods

### Study design and setting

The present study was a hospital-based case-control study conducted in the department of Gynecological Oncology of the National Institute of Cancer Research and Hospital (NICRH) from March 2022 to July 2023.

### Study participants

The sample size for this study was calculated based on the formula for estimating sensitivity and specificity: *n = z^2^p(1-p)/d^2^*, where z is the z-score corresponding to a 95% confidence level (1.96), p is the anticipated sensitivity, and d is the desired precision. For estimating sensitivity, an anticipated value of 95.2% was used, based on a prior study using the ROMA index.^20^ With 5% precision, the required number of cases was calculated to be 71. However, considering logistical constraints, a total of 100 women were enrolled in this study. Among them, 50 women aged over 18-years with a confirmed diagnosis of epithelial ovarian cancer, established through histopathological examination of surgical or biopsy specimens, were included as cases. The benign ovarian disease group comprised 25 women diagnosed with non-malignant ovarian conditions, including cystadenomas, endometriosis, and fibroma-thecomas (confirmed by histopathology), as well as functional cysts and mature teratomas diagnosed by transvaginal ultrasonography. The healthy control group consisted of 25 apparently healthy women, free from ovarian pathology, confirmed through detailed clinical assessment and baseline investigations.

Participants were recruited using a purposive sampling technique, following these predefined inclusion and exclusion criteria. Inclusion criteria for cases were women aged above 18-years with histologically confirmed epithelial ovarian cancer. For the benign ovarian disease group, eligibility required a diagnosis of benign ovarian pathology confirmed by histopathology or transvaginal ultrasonography. Healthy controls were required to have no evidence of ovarian disease or other major systemic illnesses. Exclusion criteria for all groups included a prior history of chemotherapy or hormonal therapy, previous bilateral oophorectomy, presence of serious cardiac, hepatic, or renal diseases, diagnosis of non-epithelial ovarian malignancy, and current pregnancy.

### Data collection

After obtaining informed written consent, women with a suspected pelvic mass and ovarian pathology who attended the study site were screened for inclusion criteria by detailed history, clinical examination, and relevant laboratory investigations. Those who met the inclusion criteria for cases and controls were recruited for the study, respectively. All the participants were tested for serum HE4 and CA-125 and the ROMA index was calculated. A predesigned case record form was used for each of the participants to collect data, which included all the variables of interest.

### Laboratory procedure

With aseptic precautions, a sample of 3 mL venous blood was drawn from each participant using a clot activator tube by a trained laboratory technician. Immediately after collection, the serum was separated from the whole blood through fine centrifugation and stored at −80 °C.

Serum CA-125 and HE4 level were assessed using the Chemiluminescence Immunoassay (CLIA) technique with the MAGLUMI 2000 Plus Integrated System (Snibe Diagnostic) using MAGLUMI CA-125 (Cat. n° 130201009M) and MAGLUMI HE4 (Cat. n° 130201025M) assay kits. Firstly, the serum sample was mixed with magnetic microbeads coated with anti-CA-125 monoclonal antibodies (for CA-125 assay) or anti-HE4 monoclonal antibodies (for HE4 assay) and a buffer, allowing the CA-125 and HE4 antigen in the sample to bind to the coated microbeads. Following magnetic precipitation and decanting the supernatant, a wash cycle was performed. An Amino-Butyl-Ethyl-Isoluminol (ABEI) labeled monoclonal antibody was then added and incubated to form a sandwich immuno-complex. After another round of magnetic precipitation and washing, a catalyst solution containing 1.5% NaOH and 0.21% hydrogen peroxide was added to initiate a chemiluminescence reaction. The resulting light signal was measured in Relative Light Units (RLUs) by a photomultiplier, which was directly proportional to the CA-125 and HE4 concentration in the sample.

Serum CA-125 and HE4 values were then input into the ovarian cancer risk assessment software (Abbott Diagnostics), followed by automatic calculation of the corresponding ROMA index.

The assay kits and ROMA software were procured through standard institutional channels, and all testing and calculations were performed independently by the study laboratory personnel without influence or input from the manufacturer.

The premenopausal calculation formula of the ROMA index was^21^:

PredictiveIndex(PI)=(−)12.0+2.38xLN(HE4)+0.0626xLN(CA−125)

The postmenopausal calculation formula of the ROMA index was:

PredictiveIndex(PI)=(−)8.09+1.04xLN(HE4)+0.732xLN(CA−125)

The ROMA value (predictive value) was subsequently calculated using the following equation:

ROMAvalue(%)=100xexp(PI)/(1+exp(PI)

### Statistical analysis

All statistical analyses were carried out using STATA version 17.0. Continuous variables were summarized as mean with Standard Deviation (SD) if normally distributed and as median with Interquartile Range (IQR) if non-normally distributed, while categorical variables were reported as frequencies and percentages. Differences in normally distributed continuous variables, such as age and BMI, were assessed using one-way analysis of variance (ANOVA). Non-normally distributed biomarkers were compared across groups using the Kruskal-Wallis test, with Dunn’s post-hoc test applied for pairwise comparisons between ovarian cancer, benign ovarian disease, and healthy control groups. For evaluating the diagnostic performance of these markers (CA-125, HE4 and ROMA index), sensitivity and specificity were analyzed. Receiver Operator Characteristic (ROC) curves and the Areas Under the Curve (AUC) with 95% Confidence Interval (95% CI) were calculated for each of the markers. For all statistical outputs, a p-value < 0.05 was considered statistically significant.

## Results

The mean (SD) ages of the participants with epithelial ovarian cancer, benign ovarian diseases, and healthy controls were 52.5 (8.0), 38.2 (9.6), and 45.0 (6.7) years, respectively. Postmenopausal status was predominant among ovarian cancer cases (72%), whereas most participants in the other two groups were premenopausal. Other baseline characteristics, including parity, Body Mass Index (BMI), oral contraceptive use, and family history of breast or ovarian cancer, did not differ significantly across groups (Table 1). Among ovarian cancer cases, serous carcinoma was the most common histological subtype (58%), followed by endometrioid, clear cell, and mucinous carcinomas. According to FIGO staging, 42% presented at stage I, while 30% and 18% had advanced-stage (stage III/IV) disease. Tumor grade distribution showed a similar pattern across grades I to III (Table 2). In the benign ovarian disease group, endometriosis and serous cystadenoma were most frequent (Table 3).

**Table 1:** Baseline characteristics of the participants (*n* = 100).

Characteristics	Ovarian cancer (*n* = 50)	Benign ovarian disease (*n* = 25)	Healthy control (*n* = 25)	p-value
Age	52.54 (8.02)	38.20 (9.58)	45.04 (6.67)	<0.001^a^
Menstrual status				<0.001
Premenopausal	14 (28.00)	20 (80.00)	15 (60.00)	
Postmenopausal	36 (72.00)	5 (20.00)	10 (40.00)	
Marital status				0.002
Married	48 (96.00)	17 (68.00)	20 (80.00)	
Unmarried	2 (4.00)	8 (32.00)	5 (20.00)	
Parity				0.144
≤ 2	30 (60.00)	19 (76.00)	20 (80.00)	
> 2	20 (40.00)	6 (24.00)	5 (20.00)	
BMI	22.56 (4.19)	23.67 (4.38)	24.08 (3.81)	0.224^a^
BMI category				0.632
Underweight	16 (32.00)	7 (28.00)	4 (16.00)	
Normal weight	16 (32.00)	7 (28.00)	10 (40.00)	
Overweight	18 (36.00)	11 (44.00)	11 (44.00)	
History of OCP intake				0.332
Yes	14 (28.00)	7 (28.00)	11 (44.00)	
No	36 (72.00)	18 (72.00)	14 (56.00)	
Family history of breast cancer				0.267
Yes	12 (24.00)	5 (20.00)	2 (8.00)	
No	38 (76.00)	20 (80.00)	23 (92.00)	
Family history of ovarian cancer				0.766
Yes	7 (14.00)	2 (8.00)	2 (8.00)	
No	43 (86.00)	23 (92.00)	23 (92.00)	

a One-way ANOVA.

**Table 2:** Histological characteristics, FIGO classification and grading of ovarian cancer (*n* = 50).

Characteristics	n (%)
Histology of ovarian cancer	
Serous carcinoma	29 (58.00)
Clear cell carcinoma	7 (14.00)
Endometrioid carcinoma	9 (18.00)
Mucinous carcinoma	5 (10.00)
FIGO classification of ovarian cancer	
Stage 1	21 (42.00)
Stage 2	5 (10.00)
Stage 3	15 (30.00)
Stage 4	9 (18.00)
Grading of ovarian cancer	
Grade I	18 (36.00)
Grade II	15 (30.00)
Grade III	17 (34.00)

**Table 3:** Histological characteristics of benign ovarian disease (*n* = 25).

Histology of benign ovarian disease	n (%)
Endometriosis	5 (20.00)
Fibroma-thecoma	3 (12.00)
Functional cyst	4 (16.00)
Mature teratoma	4 (16.00)
Mucinous cystadenoma	4 (16.00)
Serous cystadenoma	5 (20.00)

**Fig. 1:**
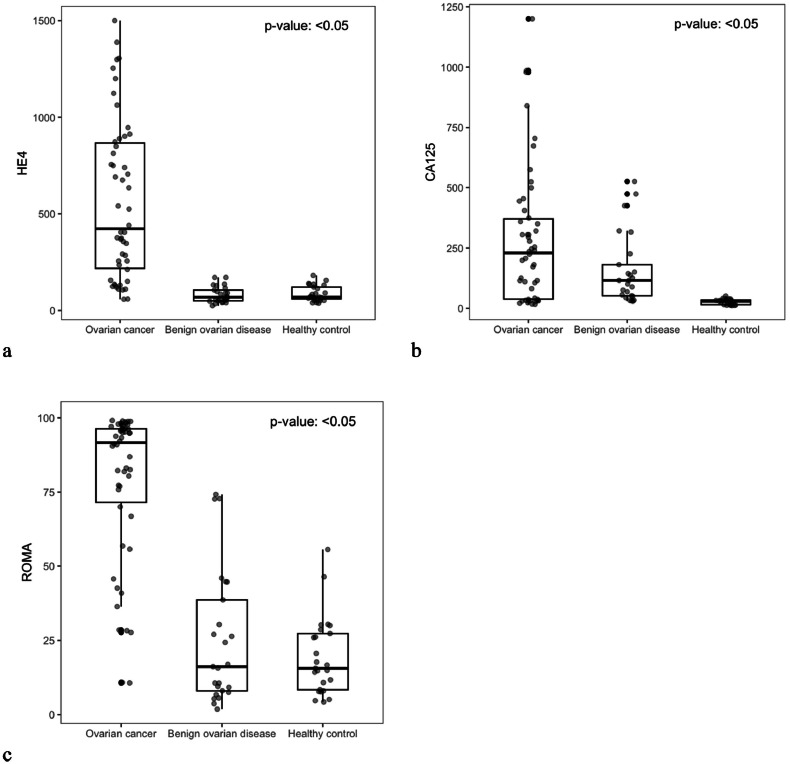
Serum level of HE4 (pmoL/L) (a), CA-125 (U/mL) (b), and ROMA (%) (c) of patients with ovarian carcinoma, benign ovarian diseases and healthy control groups. All group comparisons were statistically significant (p-value < 0.05, Kruskal-Wallis test), as were pairwise comparisons between ovarian cancer vs. benign disease and ovarian cancer vs. healthy controls (p-value < 0.05, post-hoc Dunn's test) for all indices.

The median serum levels of HE4, CA-125, and the ROMA index were significantly higher in ovarian cancer cases compared to both benign disease and healthy control groups (all p-values < 0.05) (Fig. 1).

**Fig. 2:**
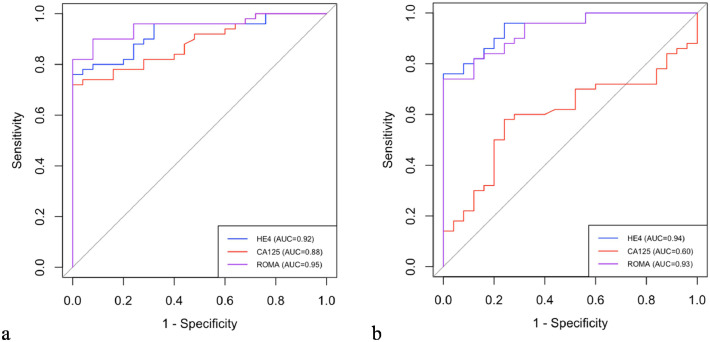
ROC curves for HE4, CA-125, and ROMA between ovarian cancer patients and healthy control group (a) and benign ovarian disease group (b).

The ROC analysis demonstrated that HE4, CA-125, and the ROMA index could effectively discriminate ovarian cancer from healthy controls, with AUCs of 0.92, 0.88, and 0.95, respectively (all *p* < 0.001). In distinguishing ovarian cancer from benign ovarian diseases, HE4 and the ROMA index remained significant (AUCs 0.94 and 0.93, *p* < 0.001), while CA-125 did not reach statistical significance (AUC = 0.60, *p* = 0.165) (Fig. 2).

Stage-stratified ROC analysis indicated that the ROMA index performed better in late-stage ovarian cancer compared to early-stage disease. When differentiating ovarian cancer from healthy controls, the AUC was 0.99 for late-stage versus 0.92 for early-stage disease. For distinguishing ovarian cancer from benign ovarian conditions, the AUC was 0.96 for late-stage versus 0.90 for early-stage disease.

The estimated optimal cut-off values for differentiating ovarian cancer from healthy controls were 126.8 pmoL/L for HE4 (86% sensitivity, 76% specificity), 33.1 U/mL for CA-125 (82% sensitivity, 72% specificity), and 33.4% for the ROMA index (90% sensitivity, 92% specificity). Cut-off values were selected to maximize Youden’s index to achieve the best balance between sensitivity and specificity. When comparing ovarian cancer to benign ovarian diseases, the respective cut-offs were 129.5 pmoL/L for HE4 (86% sensitivity, 84% specificity), 103.3 U/mL for CA-125 (70% sensitivity, 48% specificity), and 50.8% for the ROMA index (82% sensitivity, 94% specificity) (Table 4, Fig. 2).

**Table 4:** Comparison of ROC–AUCs, sensitivity, and specificity for HE4, CA-125, and ROMA index among the ovarian cancer, benign ovarian disease and healthy control group.

Marker	Cut off value	p-value	AUC (95% CI)	Sensitivity (%)	Specificity (%)
Ovarian cancer vs. healthy controls					
HE4 (pmoL/L)	126.8	<0.001	0.92 (0.87‒0.98)	86.0	76.0
CA-125 (U/mL)	33.1	<0.001	0.88 (0.81‒0.96)	82.0	72.0
ROMA (%)	33.4	<0.001	0.95 (0.91‒0.99)	90.0	92.0
Ovarian cancer vs. benign ovarian diseases					
HE4 (pmoL/L)	129.5	<0.001	0.94 (0.89‒0.99)	86.0	84.0
CA-125 (U/mL)	103.3	0.165	0.60 (0.47‒0.73)	70.0	48.0
ROMA (%)	50.8	<0.001	0.93 (0.88‒0.98)	82.0	94.0

Post hoc power analysis for the ROMA index showed that it had 62% power for a sensitivity of 90% and 79% power for a specificity of 92% to discriminate ovarian cancer from healthy controls, whereas HE4 had moderate power, and CA-125 showed limited power, consistent with their observed diagnostic performance.

## Discussion

The authors found that the ROMA index had the highest diagnostic accuracy for distinguishing epithelial ovarian cancer from benign ovarian diseases and healthy controls, followed by HE4, while CA-125 showed the lowest diagnostic performance.

In the comparative ROC analysis, the ROMA index consistently outperformed both HE4 and CA-125, achieving the highest AUC values for differentiating ovarian cancer from both healthy controls and benign ovarian diseases. Besides, ROMA showed higher AUC in late-stage vs. early-stage disease. This can be attributed to its integrated nature, combining the tumor-specific marker HE4 with CA-125 while adjusting for menopausal status, which might substantially enhance the diagnostic accuracy.^21^ Several studies have similarly reported the superior diagnostic performance of the ROMA index, with AUCs ranging between 0.91 and 0.96 in differentiating malignant from benign pelvic masses.^17^, ^19^, ^20^, ^22^, ^23^ The diagnostic accuracy of the ROMA index was higher in detecting late-stage ovarian cancer compared to early-stage disease. This finding is consistent with prior research demonstrating that serum concentrations of HE4 and CA-125 tend to rise progressively with tumor advancement, thereby improving the sensitivity of biomarker-based algorithms in advanced stages.^21^ However, post hoc power analysis indicated only 62% power for sensitivity and 79% for specificity, which is below the conventional threshold; therefore, while ROMA demonstrates superior diagnostic performance, these findings should be interpreted with caution, as limited power may reduce confidence in some estimates.

Besides, HE4 alone demonstrated a high diagnostic performance in the present study. Its significant discriminative ability of malignant ovarian disease from both healthy controls and benign ovarian diseases aligns with prior literature, where HE4 has shown improved specificity compared to CA-125.^12^^,^^24^^,^^25^ HE4 is less frequently elevated in benign gynecological conditions,^26^ which has made it a valuable adjunct in ovarian cancer diagnostics. However, despite its high AUC, HE4's specificity was slightly lower than the ROMA index in the present analysis, reflecting the advantage of combining biomarkers and adjusting for menopausal status. While ROMA outperformed HE4, the limited post hoc power highlights the need for caution in interpreting these comparisons.

In contrast, CA-125 displayed limited diagnostic value, especially when differentiating ovarian cancer from benign ovarian diseases. Its relatively low AUC in this context corroborates earlier studies that documented suboptimal specificity and sensitivity for CA-125 in ovarian cancer detection.^17^^,^^22^^,^^27^ Although widely used in clinical practice, CA-125 is prone to elevation in a variety of benign conditions, including endometriosis, pelvic inflammatory disease, menstruation, and hormone therapy etc.^28^

Regarding the cut-off values applied in this study, they were selected based on ROC-derived optimal thresholds maximizing sensitivity and specificity for differentiating malignant from benign or healthy states. Optimal cut-off values may vary between populations and assay platforms, which is a strength of this population-specific study. The HE4 cut-off value around 126–129 pmoL/L corresponds with previously established diagnostic thresholds used in multiple ovarian cancer studies,^12^ balancing high sensitivity (> 85%) with acceptable specificity. The CA-125 cut-off varied more broadly, but a value of approximately 33–103 U/mL reflects the commonly accepted clinical range, where levels above 35 U/mL often raise suspicion of malignancy, though higher thresholds improve specificity.^29^ The ROMA index cut-offs of approximately 33%–50% were chosen to reflect points of maximal diagnostic discrimination, though it was slightly higher compared to the previous reports.^19^

A notable consideration of these findings is the significant age difference among study groups. As HE4 and CA-125 levels increase with age, this imbalance may have contributed to higher biomarker concentrations in the cancer group independent of disease status, potentially inflating observed performance. While the ROMA index accounts for menopausal status, it does not fully adjust for age-related variation within pre- or postmenopausal subgroups. Consequently, these results should be interpreted with caution, and future studies should include age-matched cohorts or multivariable adjustment to better estimate biomarker accuracy.

**The present study has several limitations. First, it was conducted at a single tertiary care center, potentially limiting patient diversity and generalizability. Additionally, the case-control design introduces inherent selection bias, as participants with known ovarian pathology were selectively enrolled.** The relatively modest sample size also limits the precision of cut-off estimations and subgroup analyses and limits the statistical power for HE4 and CA-125. The post hoc analysis showed that the study was underpowered for some comparisons, which might be a consequence of the smaller-than-planned sample size, and this makes some performance estimates unstable and should be interpreted cautiously. In the present study, ovarian cancer cases were considerably older than participants with benign ovarian conditions or healthy controls. Because both HE4 and CA-125 levels tend to increase with age, this age difference may have affected the observed biomarker concentrations and, consequently, their apparent diagnostic performance in distinguishing malignant from non-malignant cases. Although the ROMA index incorporates menopausal status in its calculation, it does not fully adjust for age-related variations within pre- or postmenopausal subgroups in this population. Stratified analyses for HE4 and CA-125 could not be performed due to the limited sample size, as dividing the cohort further would have reduced statistical power. These factors represent potential confounders that should be carefully considered when interpreting the present findings. Future studies in Bangladeshi women should aim to use age-matched groups or apply multivariable adjustment and, where possible, perform stratified analyses by age and menopausal status to provide more robust and generalizable estimates of biomarker accuracy. **An important consideration in interpreting the findings of the study would be the potential factors influencing biomarker performance, like age, menopausal status, renal function, and comorbid conditions.** While menopausal status was considered in the ROMA calculation, other variables were not adjusted in this study, which might have impacted diagnostic accuracy. **Moreover, as HE4 can be elevated in patients with impaired renal function, and CA-125 in various inflammatory or physiological conditions, accounting for these factors in future analyses would improve clinical applicability.**^30^ **Moreover, the diagnostic performance of these markers was not validated in an external population, which is essential for confirming their clinical utility. Finally, this was a case-control study, which may be valid for initial biomarker evaluation, but can overestimate diagnostic accuracy compared to a real-world cohort of consecutive patients with pelvic masses.**

Future research should prioritize multi-center studies with larger, diverse populations to externally validate these findings. Additionally, evaluating these biomarkers in asymptomatic women at high risk for ovarian cancer or those with indeterminate pelvic masses would offer valuable insights into their screening potential. Longitudinal studies assessing biomarker levels in relation to treatment response, disease recurrence, and overall survival could further clarify the prognostic value of these biomarkers. Integrating these markers into risk prediction models incorporating clinical features, imaging, and genetic profiles could enhance personalized management strategies for ovarian cancer.

## Conclusions

In conclusion, these findings suggest that the ROMA index may provide better diagnostic performance than HE4 and CA-125 alone for distinguishing epithelial ovarian cancer from benign pelvic masses. However, the observed differences should be interpreted cautiously, as this case-control study may not fully reflect real-world performance. Its combination of biomarker levels with menopausal status could contribute to improved risk assessment, but these results require confirmation in larger, prospective cohorts. The authors suggest that the ROMA index could be explored further in diagnostic pathways, particularly in resource-limited settings like Bangladesh, with the suggestion that additional validation is needed before widespread clinical use. Future research should include multi-center studies in South Asian populations, integration with imaging modalities such as transvaginal ultrasonography, and evaluation of prognostic implications of biomarker trends for predicting treatment response, recurrence, and survival.

## Abbreviations

CA, Carbohydrate Antigen; HE, Human Epididymis; ROMA, Risk of Ovarian Malignancy Algorithm; NICRH, National Institute of Cancer Research and Hospital; ABEI, An Amino-Butyl-Ethyl-Isoluminol; RLUs, Relative Light Units; SD, Standard Deviation; ROC, Receiver Operator Characteristic; CI, Confidence Interval; BMI, Body Mass Index; AUC, Area Under the Curve.

## Ethics approval and consent to participate

The study received ethical permission from the institutional review board of Institutional Review Board (IRB) of BIRDEM General Hospital (n° BIRDEM/IRB/2022/337). This study was conducted as part of the MD thesis work of P.K.D., who was an enrolled trainee under the academic supervision of BIRDEM General Hospital. Accordingly, ethical approval was obtained from the IRB of BIRDEM. However, prior to data collection, formal administrative permission was also obtained from the National Institute of Cancer Research and Hospital (NICRH), where patient recruitment and data collection were carried out. Informed written consent was taken from the study subjects. All authors declare no human subjects were harmed and the procedures followed were in accordance with the ethical standards and regulations established by the Helsinki Declaration of the World Medical Association.

## Consent for publication

All authors agree to publish the article.

## Funding

The authors have no support or funding to report.

## CRediT authorship contribution statement

**Pallab Kumar Das:** Conceptualization, Formal analysis, Methodology, Resources, Supervision, Writing – original draft. **Saddam Hossain:** Conceptualization, Formal analysis, Investigation, Resources, Writing – original draft. **A.F.M. Ashrafur Rahman:** Conceptualization, Formal analysis, Investigation, Writing – original draft. **Pritam Saha:** Conceptualization, Investigation, Methodology, Writing – original draft. **Shaker Ahmed:** Conceptualization, Investigation, Methodology, Writing – original draft. **Tauhid Md Mustakim:** Conceptualization, Formal analysis, Investigation, Methodology, Writing – original draft. **Kazi Fharia Tanjim:** Conceptualization, Formal analysis, Methodology, Writing – original draft. **Asesh K. Chowdhury:** Conceptualization, Formal analysis, Investigation, Resources, Supervision, Writing – original draft. **Tawfika Rahman Jishan:** Conceptualization, Formal analysis, Methodology, Resources, Writing – original draft. **Tanjina Tabassum:** Conceptualization, Formal analysis, Investigation, Methodology, Resources, Supervision, Writing – original draft.

## Declaration of competing interest

The authors declare no conflicts of interest.

## Data Availability

Patient-level data will be available on request from the corresponding author.

## Appendix Supplementary materials

Image, application 1
